# Neuronal Representation of Auditory Distance Percepts vs. Cues in Human Auditory Cortex

**DOI:** 10.1007/s10162-025-01026-8

**Published:** 2026-01-30

**Authors:** Keerthi Kumar Doreswamy, Jyrki Ahveninen, Samantha Huang, Stephanie Rossi, Norbert Kopčo

**Affiliations:** 1 Athinoula A. Martinos Center for Biomedical Imaging, Department of Radiology, Harvard Medical School/Massachusetts General Hospital, Charlestown, MA 02129 USA; 2 Institute of Computer Science, P. J. Šafárik University, Košice, 04001 Slovakia

**Keywords:** Auditory distance perception, FMRI, MVPA, DRR, Neural coding, Auditory cortex

## Abstract

**Purpose:**

Perceiving the sound source distance is important in many everyday activities. For sources near the listener, two dominant intensity-independent cues are available, the direct-to-reverberant energy ratio (DRR) and the interaural level difference (ILD). Previous studies identified the planum temporale (PT) and posterior superior temporal gyrus (pSTG) as auditory cortical areas important for distance processing. However, it is not clear whether the identified areas represent integrated percepts of distance, per se*,* or the discrete cues based on which it is created. To address this, we combined behavioral and neuroimaging experiments with advanced computational modeling.

**Methods:**

We conducted human behavioral (15 participants with 5 self-reported females) and fMRI experiments (15 participants with 5 self-reported females) in a virtual reverberant environment using broadband noise stimuli. The availability and congruency of the DRR and ILD cues were manipulated to identify cortical areas encoding distance cues vs. distance percepts.

**Results:**

Behavioral results showed that distance percepts were stronger when both cues were available and congruent, confirming that both cues are used when listeners judge distance. A univariate fMRI analysis identified areas in the PT + pSTG as encoding the DRR cue. An ROI-based multi-voxel pattern analysis (MVPA) over the whole PT + pSTG region found a significant difference between Congruent and Incongruent stimuli, likely representing the distance percept.

**Conclusion:**

The PT + pSTG region encodes both the distance cues and percepts. However, while the cue encoding is local, the percepts are encoded in a distributed network.

## Introduction

In many everyday activities, judging the distance of sound sources is important [1–5]. Consider, for instance, avoiding an approaching car outside our field of vision [6, 7] or trying to hear out a nearby talker in a noisy environment [1, 8–10]. Although auditory distance perception is critical in such scenarios, its functional mechanisms and neural basis are not well understood [3, 5, 11, 12].

The main cue for distance perception is often considered to be the received sound intensity [13]. However, the intensity with which a sound is emitted is often variable or a priori unknown. In such situations, listeners naturally use other cues. Multiple intensity-independent distance cues are often available [3]. Kopco et al. [14] studied two intensity-independent distance cues. First, the direct-to-reverberant energy ratio (DRR), which compares the sound energy received at the ears directly from the source to that reflected off the walls, can be used to determine the distance for all directions but only in reverberant environments [15–17]. Second, the interaural level difference (ILD) [8, 18] can be used in both reverberant and anechoic environments, but only for sources off the midline. These cues are robust particularly when the sources are in the peri-personal space (up to 1–2 m from the listener) where the listener can interact with the sound sources [3, 19]. In addition to the primary cues of ILD and DRR, multiple other distance cues are also available, such as spectrum, binaural parallax, vocal effort, interaural cross-correlation (IACC) [5] and amplitude and spectral modulation [20–22]. Importantly, while the DRR cue is considered as the reverberation-related cue here, most of the reverberation-related cues mentioned like frequency-to-frequency variation in the spectrum [23], amplitude modulation reduction [24], and IACC are highly correlated and, hence, any of them might be the actual cue used by the listeners. Thus, we consider DRR as the representative cue for its convenience and clarity of computation while being largely agnostic as to which reverberation-related cue is actually used by the listeners. Finally, even though the mechanism by which the distance cues are integrated to create a distance percept has not been established, it is likely that the weight with which they are combined depends on the target azimuth, distance, type of stimulus, the presence of room reverberation, the type of task (absolute vs. relative localization), as well as non-auditory factors. Hence, the cues are likely combined in a context-dependent way, making the mechanisms underlying auditory distance perception difficult to unentangle [25–28].

Some of the distance cues are shared between distance and other spatial dimensions. Specifically, the ILD is also a horizontal cue, which for frontal sources varies primarily with their azimuth, while for lateral nearby sources it varies primarily with distance [19]. Also, monaural spectral changes, which are the primary elevation cue, can also provide monaural azimuthal information, as well as distance information in reverberation [29]. Important for the current study, the monaural spectral distance cue highly correlates with DRR; therefore, it can be thought of as one of the reverberation-related cues included when DRR is considered [23, 24]. The current study focuses only on distance perception of nearby sources along the interaural axes, where the ILD and DRR cues strongly vary with distance.

Considering how the intensity-independent distance cues are combined, Kopčo and Shinn-Cunningham [30] proposed that DRR is the primary distance cue in reverberation, based on acoustic analysis and modeling of behavioral performance in an experiment in which azimuth as well as distance were manipulated. However, a later analysis carried out on data for sources at various distances in a single direction along the interaural axis indicated the employment of both ILD and DRR cues [14], while later it has been shown that, for frontal stimuli, the distance judgements are indeed primarily based on the DRR [31]. This study focuses only on DRR (as a representative reverberation-related cue) and ILD, aiming to understand how these intensity-independent distance cues are encoded and integrated to create intensity-independent distance percepts. 1

The neural basis of auditory spatial perception is understood less than its psychophysics. In human neuroimaging studies, only relatively broad anatomical divisions have been found, such as that between the anterior “what” vs. posterior “where” pathways [32–36]. A majority of the spatial neuroimaging studies focused on the encoding of the horizontal sound location (i.e., of the location percept) or on the encoding of the binaural cues that underlie it [37–44]. Activations were found primarily in the posterior auditory “where” pathway, which includes the non-primary auditory cortex regions planum temporale (PT) and posterior superior temporal gyrus (pSTG) [45, 46]. Kopco et al. [14] performed an fMRI study to identify the brain representation of distance independent of intensity for nearby stimuli varying in distance along the interaural axis. They identified a distance representing region in the pSTG and PT of the hemifield contralateral to the stimulation, likely encoding distance primarily based on ILD and DRR. Using similar methods and stimuli presented in the frontal direction, Kopco et al. [31] examined distance representations that do not involve the ILD cue used also for horizontal localization. They found the AC region encoding DRR-based distance information, encompassing bilaterally the posterior banks of Heschl’s gyri, PT, and pSTG. These findings imply that neuronal populations sensitive to distance independent of intensity exist in the posterior auditory cortex. Still, the question of whether those identified brain regions represent the distance percept per se or one of the acoustic distance cues, e.g., ILD or DRR, needs to be answered.

The studies of Kopco et al. [14, 31] used univariate fMRI analysis to identify the individual voxels encoding distance information. Multi-voxel pattern analysis (MVPA) [47, 48] looks at the information distributed across groups of voxels, where the activation levels of each voxel alone may be less informative. As a result, they are sensitive to distributed encoding of information that is likely common in the brain. Previous studies [42, 48–51] have been successful in using multivariate methods to identify the neural correlates of various perceptual effects inaccessible to the standard univariate methods. In the current study, we use univariate and multivariate methods to identify both local encoding and encoding in distributed networks.

We performed one behavioral and one imaging experiment in a virtual reverberant environment for nearby sources along the interaural axis. We manipulated the Binaural Room Impulse Responses (BRIRs) [52] used to synthesize stimuli so that they provide different amounts of distance information. Specifically, we modified the original BRIRs, in which DRR and ILD varied with distance congruently, to create stimuli in which the cues varied incongruently, and stimuli in which only the ILD cue was available while the DRR value was fixed independent of distance. In the behavioral experiment, distance discrimination performance was measured for stimuli with Congruent, Incongruent, and Fixed-DRR cues. It was expected that if both cues are used, then presenting the cues incongruently would result in a weakening of the distance percept compared to congruent presentation, and Fixed-DRR performance would be better than Incongruent and worse than Congruent performance. In the imaging experiment, we used a sparse-sampling adaptation fMRI paradigm [32] to measure activations evoked by the same three types of stimuli. The conditions with Congruent and Incongruent cues were key for distinguishing between representations of the cues vs. the representations of the distance percept itself. Specifically, if a brain region is activated for Congruent but not for Incongruent cues, then it was expected to represent the distance percept, as both cues were available in both conditions. On the other hand, a region that is activated by both Congruent and Incongruent cues is more likely to represent one or both cues. A comparison of the Fixed-DRR responses to Congruent or Incongruent responses was expected to primarily indicate regions encoding DRR, as this cue was not available in the Fixed-DRR stimuli. The emitted stimulus intensity was fixed in the imaging experiment, consistent with previous studies [14, 31]. 2 All stimuli were presented from the left to complement previous results [14] which only used stimuli on the right. Univariate and multivariate analysis of the fMRI data was used to identify both local and distributed neural encoding of the cues/percepts.

## Methods

Experimental procedures and equipment used in this study were similar to our previous studies [8, 14, 31] unless specified otherwise.

### Participants

Fifteen participants (5 self-reported as females, ages 21–43 years) with normal hearing (audiometric thresholds within 20 dB) participated in the behavioral experiment. A separate sample of 15 right-handed participants (5 self-reported as females, ages 22–55 years) with self-reported normal hearing participated in the fMRI experiment. Using the Tukey’s outlier detection test on the across-condition average data from the behavioral experiment, two participants were identified as outliers and excluded from further analysis (their performance was above that of the remaining participants, and both were lab members with extensive prior experience with psychoacoustic experiments). Similarly, two participants were excluded from the fMRI experiment (one fell asleep during the experiment and one had excessive head movements).

### Ethics Approval

The behavioral experiments were approved by the P. J. Šafárik University (UPJŠ) Ethical Committee. The imaging experiments were approved by the Partners Human Research Committee, the MGH Institutional Review Board. All participants gave written informed consent.

### Stimuli

**Fig. 1:**
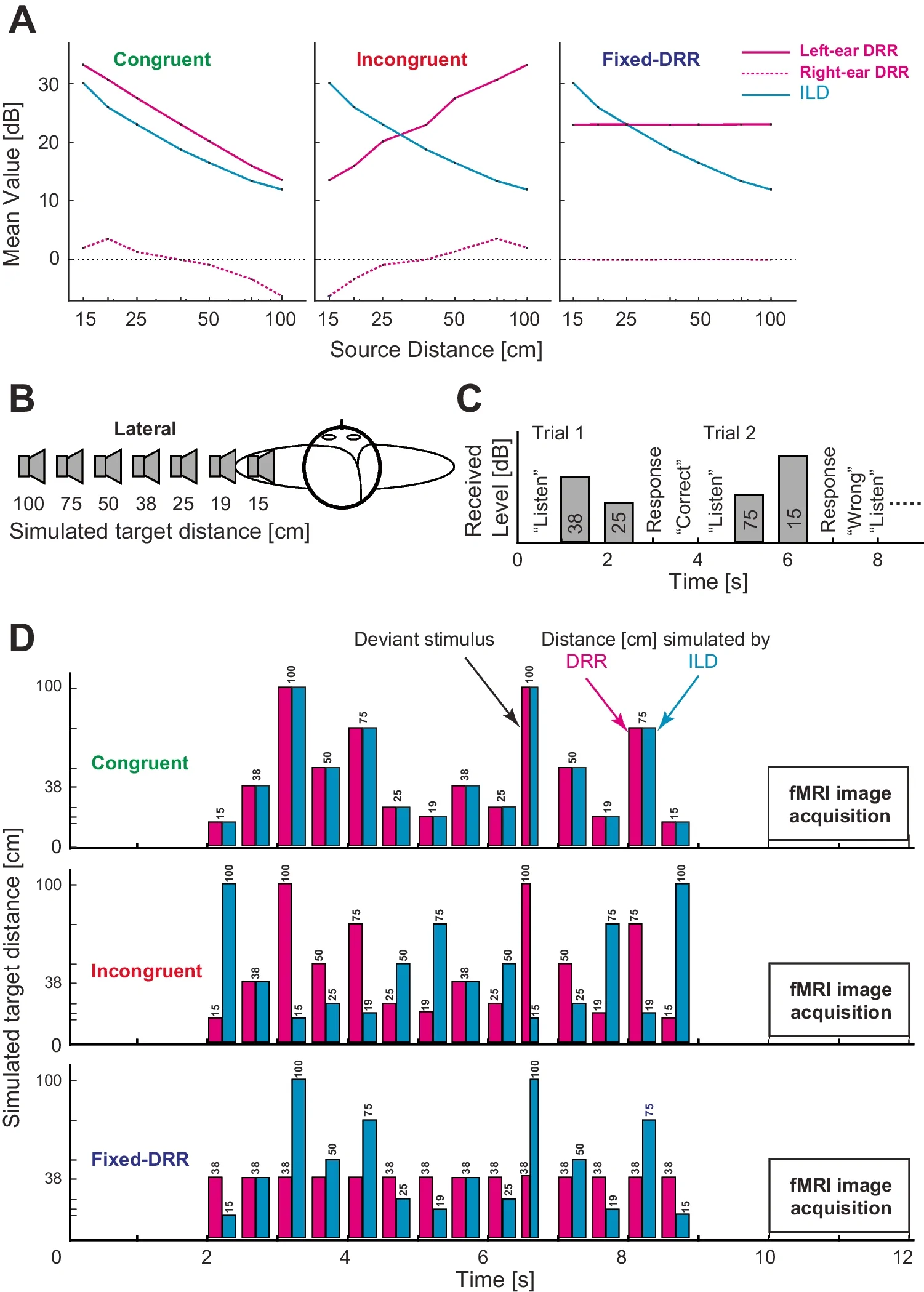
Experimental design. **A** Cue manipulation in the three stimulus conditions. In the Congruent condition, the cues ILD and DRR varied congruently with distance. In the Incongruent condition, the DRR cue values were reversed with respect to distance at both ears. In the Fixed-DRR condition, the DRR cue values were fixed at the values corresponding to a distance of 38 cm. **B** Simulated source locations along the interaural axis (left side of the listener). **C** Design of trials in the behavioral experiment: The instruction “Listen” appeared on the screen, followed by the presentation of two stimuli from different distances at random levels. Listeners responded by indicating whether the second stimulus was closer or more distant than the first stimulus. On-screen feedback was provided in some conditions. **D** Design of a single imaging trial in the fMRI experiment, shown separately for the three stimulus conditions (as shown in **A**). The height of the stimulus bars corresponds to the distance simulated by the respective cue value. In these conditions, the listener’s task was to detect deviant stimuli that were shorter than the standard stimuli. No feedback was provided. A Quiet stimulus condition was also included (not shown)

The auditory distance stimuli were generated using a single set of non-individualized BRIRs measured on an individual not participating in the current study using methods identical to Shinn-Cunningham et al. [8]. The BRIRs were measured in a small-sized carpeted room (reverberation times T_60_ ranging from 480 to 610 ms in octave bands centered at 500, 1000, 2000, and 4000 Hz) using a surface-mount cube speaker (Bose FreeSpace 3 Series II, Bose, Framingham, MA). Miniature microphones (Knowles FG-3329c, Itasca, IL) were placed at the blocked entrances of the listener’s ear canals, and the loudspeaker was positioned at distances of 15, 19, 25, 38, 50, 75, or 100 cm from the center of the listener’s head at the level of the listener’s ears (Fig. 1B) on the listener’s left-hand side along the inter-aural axis.

The recorded BRIRs were altered to generate the three types of stimuli (Fig. 1A). The unaltered BRIRs were used for the Congruent stimuli in which both DRR and ILD varied congruently with distance. For the Incongruent and Fixed-DRR stimuli, the BRIRs were split before the arrival of the first reflection to separate the Direct (D) and Reverberant (R) portions. The R portion was then scaled separately at each ear so that the broadband DRR of the modified stimuli was either constant at the value corresponding to 38 cm (for the Fixed-DRR stimuli) or matched the DRR of a “reflected” distance (for the Incongruent stimuli)—e.g., the 15 cm stimulus would have the DRR of the 100 cm stimulus, etc. Finally, the overall RMS level was scaled to match that of the original stimuli. Since the frequency spectrum of the R portion does not have a strong distance dependence, and since ILD was not modified, there was no need for any frequency-dependent processing.

A set of 50 independent noise burst tokens of 300-ms white-noise samples, bandpass-filtered by zeroing the off-frequency components (i.e., applying a rectangular window in the frequency domain) in the frequency domain at 100–8000 Hz, was convolved with each of the BRIRs to generate standard stimuli for each source distance. For the fMRI experiments, an identical set of 150-ms deviant stimuli were also created. For each experimental trial, either two (behavioral experiment, Fig. 1C) or 14 (imaging experiment, Fig. 1D) noise bursts were randomly selected, scaled (see below), and placed in a series with a fixed stimulus onset asynchrony (SOA), to create the stimulus sequence. The only difference between the behavioral and imaging experiments was in the intensity normalization used in the scaling step. In the behavioral experiment, each noise burst was normalized so that its overall intensity received at the left (closer) ear was fixed and then randomly roved over a 12-dB range so that the monaural overall intensity distance cue was eliminated at both ears (Fig. 1C). In the fMRI experiment, the stimulus presentation level was fixed, congruent with ILD. Finally, the fMRI stimuli were filtered to compensate for the headphone transfer functions by filtering the original stimuli using headphone-specific equalization filters provided with the Sensimetrics S14 headphones by the manufacturer. All stimuli were pre-generated offline at a sampling rate of 44.1 kHz. The average received level was 65 dBA (measured at the left ear of a KEMAR manikin equipped with the DB-100 Zwislocki Coupler and the Etymotic Research ER-11 microphones).

### Behavioral Experiment

The behavioral experiment was performed in the Perception and Cognition Lab at UPJŠ. The participants were seated in a double-walled sound-proof booth in front of an LCD display and a keyboard connected to a control computer, placed outside the booth, running a Matlab script to control the experiment. Fireface 800 sound processor (RME) and Etymotic Research ER-1 insert earbuds were used to play the pre-generated stimuli. Each participant completed a single, one-hour session that included two runs with the 3 stimulus types in a random order. Each run consisted of 84 trials, corresponding to 4 repetitions of 21 randomly ordered trials (one trial for each combination of 2 out of the 7 distances). The phrase “Listen” appeared on the computer screen to begin each trial, which was then followed after 200 ms by two noise tokens that were simulated to come from two different distances and had a SOA of 1000 ms (Fig. 1C). The participant was instructed to identify which of the two sounds was nearer by pressing one of two keyboard keys (“1” or “2”). Feedback for Congruent and Fixed-DRR conditions was given following the response. The experiment was self-paced, and each trial lasted, on average, 5 s. The experiment started with a practice session consisting of one Congruent run, performed before the experimental runs.

### Imaging Experiment

The imaging experiment was performed at the Martinos Center for Biomedical Imaging at MGH. The participants participated in one 2-h session, consisting of preparation, training, fitting of headphones, and structural and functional acquisitions. The control computer, running a Presentation (Neurobehavioral Systems) experimental script, presented the sounds through the Fireface 400 sound processor (RME), Pyle Pro PCA1 amplifier, and Sensimetrics S14 (Sensimetrics, Gloucester, MA) MRI-compatible headphones. Responses were collected via MRI-compatible five-key universal serial bus (USB) keyboard. A video projector was attached to the control computer and projected the instructions to the participant in the scanner. Two runs of 96 trials were performed. Each trial consisted of a 10-s stimulus presentation during which the scanner was silent, followed by 2 s of fMRI image acquisition (Fig. 1D). Trials with Congruent, Incongruent, Fixed-DRR, and Quiet (no-sound) stimuli were randomly interleaved. Each non-quiet stimulus was a sequence of 14 noise bursts that had a 500 ms SOA.

The sequences contained two noise bursts for each of the seven distances, ordered pseudo-randomly such that each distance was present at least once before the repetition of any of the distances. In 50% of the sequences, one randomly chosen burst out of the 14 bursts was replaced by a 150-ms deviant. The listener’s task during the fMRI session was to identify the deviants by pressing a button on the keyboard. This choice was made on purpose to remain consistent with our previous studies [14, 31] and to prevent participants from focusing on perceived distance. Otherwise, participants might have exerted more effort in conditions where distance was harder to perceive, and this increased attention/effort could have altered the neural response—reflecting effort rather than the neural coding of distance, which we aimed to avoid. Using a 3 T 32-channel coil, whole-head fMRI was obtained (Siemens TimTrio, Erlangen, Germany). We used a sparse-sampling gradient-echo blood oxygen level dependent (BOLD) sequence (TR/TE = 12,000/30 ms, 9.82 s silent period between acquisitions, flip angle 90°, FOV 192 mm) with 36 axial slices aligned along the anterior–posterior commissure line (3-mm slices, 0.75-mm gap, 3 × 3 mm^2^ in-plane resolution). The coolant pump was turned off while the acquisitions were being made. A multi-echo MPRAGE pulse sequence (TR = 2510 ms; 4 echoes with TEs = 1.64 ms, 3.5 ms, 5.36 ms, 7.22 ms; 176 sagittal slices with 1 × 1 × 1 mm^3^ voxels, 256 × 256 mm [2] matrix; flip angle = 7°) was used to combine anatomical and functional data to produce T1-weighted anatomical images.

### Data Analysis

#### Behavioral Data

In the distance discrimination behavioral experiment, the proportion of correct responses *P*_*C*_ was measured for each stimulus condition, distance pair, and participant. To estimate each participant’s distance sensitivity across all distance pairs in a given condition, a simple decision theory model [14] was used, modified to estimate the *nominal* sensitivity index $d_N^{\prime}$, defined as the sensitivity for discriminating the distance of two sources with a log-distance separation of 1 (i.e., distance ratio of 2.72). 3The model predicts *P*_*C*_ for two sources at distances of ${s}_{1}$ and ${s}_{2}$ as a function of the sensitivity *d′* for a given distance ratio [53]: For the model, the goodness of fit is reported as the average of the coefficient of determination *R*^2^ computed for individual participant fits:

$$P_C=\frac1{\sqrt{2\pi}}\int_{-\infty}^\frac{d'}2e^{\frac{-t^2}2}dt,\text{ where } d' = d'_N.\left|\ln{s_2}-\ln{s_1}\right|.$$

$$d^{\prime}{=d}_N^{\prime}.\left|\mathrm{ln}s_2-\mathrm{ln}s_1\right|$$

In the duration discrimination task performed during the imaging experiment, the response was classified as a hit (correct detection) if it occurred within 2.5 s after the deviant onset. We determined the hit rates (HR) and reaction times (RT) to correctly detected targets and compared the group averages of these measures across the three stimulus conditions.

Statistical comparisons of the behavioral data were done using repeated measures ANOVAs after checking for normality and sphericity using CLEAVE [54] and MATLAB. The reported statistical values were corrected for potential violations of sphericity using the Greenhouse–Geisser epsilon, followed by post hoc pairwise comparisons with Bonferroni correction. The effect sizes are reported as well.

#### fMRI Data

Cortical surface reconstructions and data were preprocessed in native space without smoothing and per session using Freesurfer 5.3. Further, the individual functional volumes were motion corrected and used as an input for the first-level volumetric statistical analysis using FSL. The task conditions were entered into a first-level general-linear model (GLM) as explanatory variables. The resulting contrast effect size estimates were co-registered to the Montreal Neurological Institute (MNI) 152 standard brain representation (2 × 2 × 2 mm^3^ resolution) for a volumetric univariate GLM group analysis. The group univariate GLM analysis was performed using the Functional Magnetic Resonance Imaging of the Brain’s (FMRIB) Local Analysis of Mixed Effects 1 (FLAME1) approach. The results of the volumetric group analyses were visualized using FSL.

#### MVPA Analysis

We used the split-half correlation [48] MVPA as implemented in the MVPA toolbox CoSMoMVPA [47]. We considered a priori regions-of-interest (ROI) PT + pSTG, which were based on the cortical labels that were used in our previous study [14]. One ROI was defined in each hemisphere by combining two anatomical FreeSurfer standard-space labels encompassing the PT and posterior aspects of the STG, which were individually resampled to each participant’s native functional space for the MVPA. In each participant, we first split the data into two halves (i.e., Run-1 as the first half and Run-2 as the second half) and estimated the response for each non-quiet category, voxel, and half separately. This analysis tests the hypothesis that the correlation between pattern vectors, obtained from the cope/beta statistics of each voxel in the ROI, is more consistent across the same-condition run pairs vs. the different-run pairs (e.g., that correlations for [Run-1 and Run-2 for Congruent sound condition] and [Run-1 and Run-2 of Incongruent sound condition] are more consistent than correlations for [Run-1 of Congruent and Run-2 of Incongruent sound condition] and [Run-1 of Incongruent, Run-2 of Congruent sound condition]). Three 2 × 2 correlation matrices were produced as a result of the correlation between each pattern in one half and each pattern in the other (one for each sound condition pair). A Fisher transformation was applied to normalize the correlations. The values in these correlation matrices were weighted either positively or negatively depending on whether they are on the diagonal (matching condition across the two halves) or not. This resulted in a weighted correlation matrix. The presence of target-specific information in the patterns is indicated if the sum for contrast pairs in this matrix is positive. For the group results, the non-parametric Wilcoxon test was run on the matrix sum values obtained for each participant and corrected for multiple comparisons.

## Results

### Behavioral Experiment

**Fig. 2:**
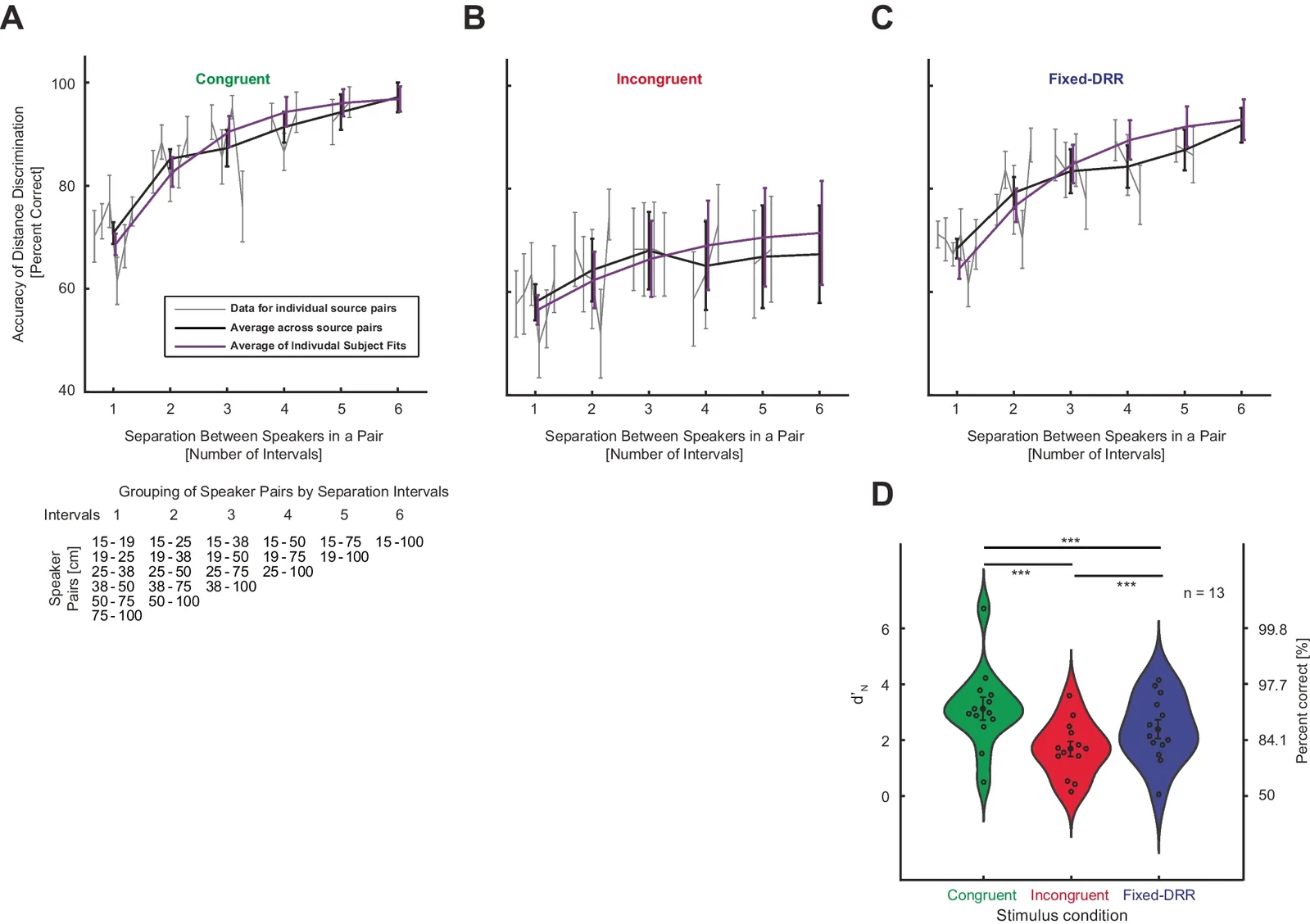
**A**,** B**,** C** Behavioral distance discrimination responses for the three stimulus conditions. The black line shows across-participant average accuracy collapsed across simulated source pairs separated by the same number of unit log-distance intervals. The purple line represents the averages of individual participant’s accuracy predictions on the basis of participant’s individual estimates of nominal distance sensitivity (${d}_{N}^{\prime}$). Grey error bars show across-participant average performance separately for each source-distance pair. They are grouped on the basis of the number of intervals between sources within the pair. Table below panel **A** lists in each column the source pairs that are separated by the same number intervals; separation ranges from 1 interval (all possible pairs of adjacent sources) to 6 intervals (the closest vs. farthest source). In each group, they are ordered from left to right based on absolute distances (listed from top to bottom in the table, respectively). Error bars represent SEM. **D** Discrimination sensitivity (lefthand *y*-axis) and percent correct accuracy for a nominal distance ratio of 2.72 (right-hand axes) for the three stimulus conditions in the behavioral experiment. The across-participants sensitivity index ${d}_{N}^{\prime}$ was estimated from responses in all 21 distance pairs in a given condition using a simple decision theory model. Black circles represent individual participants. Black error bars represent SEM. Horizontal lines and asterisks link conditions with performance significantly different in pairwise t-tests after Bonferroni correction (* for *p* < 0.05; ** for *p* < 0.01; *** for *p* < 0.005)

Figure 2A–C shows, for each condition, the percent correct discrimination performance as a function of separation in each distance pair, either averaged across the speaker pairs corresponding to the same interval (thick black line) or separately for each speaker pair (thin grey line). The data show that performance improved systematically with separation, while no systematic trend of dependence on specific speaker pair was observed, consistent with our previous studies. In addition, the across-participant average of individual model fits for each condition is shown in purple, in which the fitted values of ${d}_{N}^{\prime}$ are shown in panel D. The across-participant average goodness of fit (*R*^2^) values of the model are 0.37, 0.14, 0.19 for conditions Congruent, Incongruent and Fixed-DRR, respectively, mostly dominated by the noise in the individual participant data (as the average data and model are very close to each other). Figure 2D shows the intensity-independent distance discrimination sensitivity for the three stimulus conditions of the behavioral experiment, obtained from the fits in Fig. 2A–C. The nominal sensitivity index ${d}_{N}^{\prime}$ was lower in the Incongruent than in Congruent condition (1.79 vs. 3.36), while falling between the Congruent and Incongruent conditions for the Fixed-DRR condition (2.59). Confirming this, a repeated measures ANOVA found a significant main effect of condition (*F*_2,24_ = 6.83, *p* = 0.004; *η*^2^_G_ = 0.2; Mauchley’s test: *χ*^2^(2) = 0.21, *p* = 0.89), followed by post hoc pairwise comparisons with Bonferroni correction with significant differences for all three condition pairs. These results are consistent with the hypothesis that both DRR and ILD are used as intensity-independent distance cues, contributing significantly to the distance percept, when both are available. Specifically, decreased performance in the Fixed-DRR condition compared to the Congruent condition means that the Fixed-DRR cue in this condition made the discrimination more difficult. Most important for the current study, the Incongruent stimuli were discriminated much less accurately than the Congruent stimuli. 4

### fMRI Experiment

Behavioral and fMRI data were collected during the fMRI experiment. First, the behavioral results are presented, followed by two fMRI data analyses. The behavior was monitored to assure that the fMRI data were not contaminated by fluctuations in attention and alertness during the scanning. The behavioral task was to identify duration deviants that appeared randomly during the fMRI trials. An analysis of reaction times (RT) and hit rates (HR) was performed. The task difficulty was approximately equal across all conditions (across-participant average HRs of 77.56 ± 21.8%, 82.69 ± 22.4%, and 83.97 ± 24.3% and RTs of 1224 ± 101, 1267 ± 128, and 1263 ± 72 ms, respectively, for the Congruent, Incongruent, and Fixed-DRR conditions). Repeated measures ANOVAs performed on the RAU transformed [55] HRs and RTs found no significant differences (HR: *F*_2,24_ = 2.26, *p* > 0.1; *η*^2^ = 0.15 RT: *F*_2,24_ = 2.51, *p* > 0.1; *η*^2^ = 0.17).

**Fig. 3:**
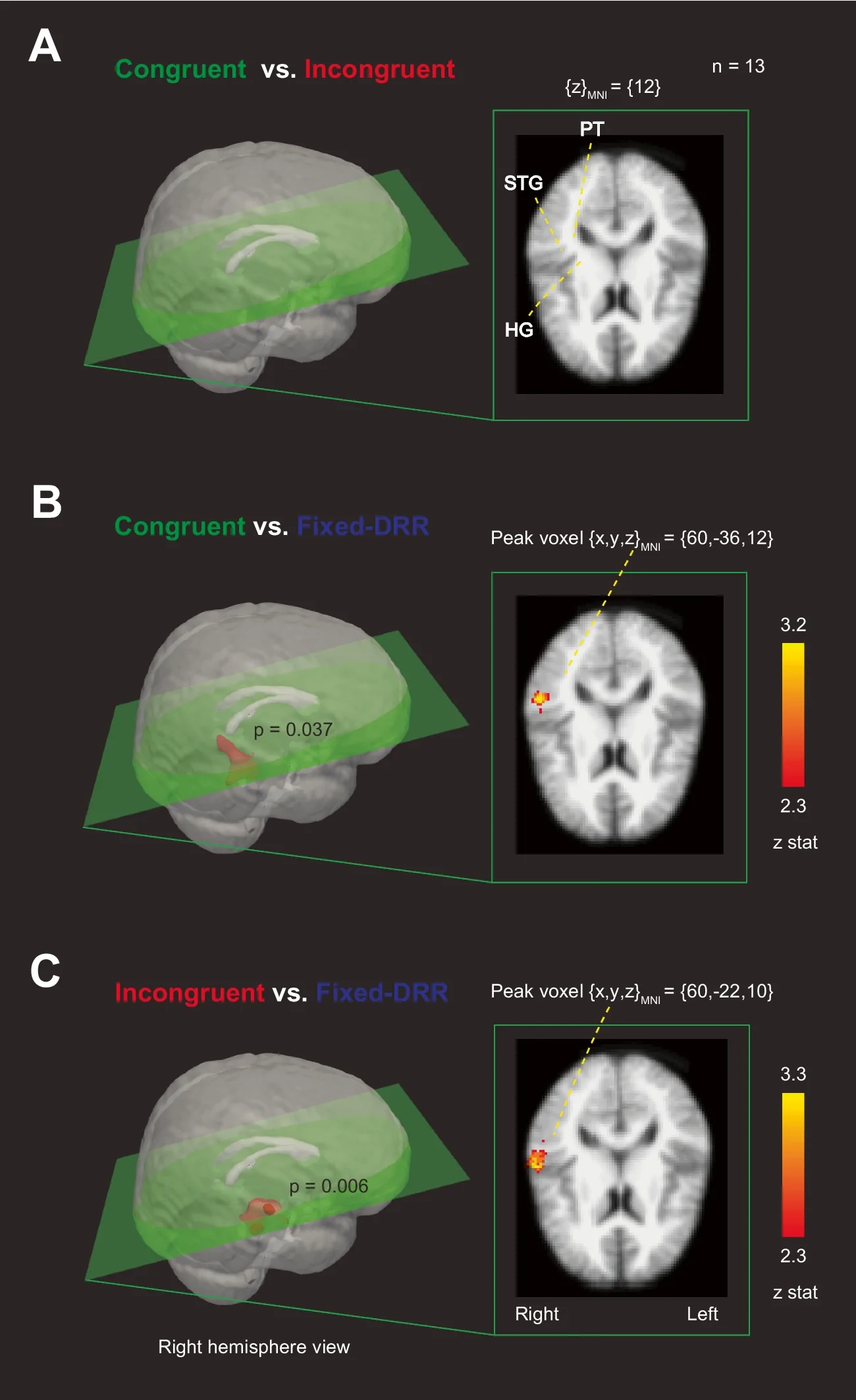
Volume-based (MNI152) fMRI thresholded activation images for different stimulus condition pairs (clusters determined by *Z* > 2.3 and a (corrected) cluster significance threshold of *p* ≤ 0.05 [88]). **A** Congruent vs. Incongruent. **B** Congruent vs. Fixed-DRR. **C** Incongruent vs. Fixed-DRR. The left panels show the significant volume clusters in the right hemisphere contralateral to the stimulus direction that extend from the PT to the posterior STG embedded into the respective lateral views of a “glass brain” representation. Right panels show horizontal plane at *z*_MNI_ coordinate including the largest activation voxel following the radiological convention

To identify cortical areas involved in distance-related processing at the level of individual voxels, we performed a volume-based fMRI univariate analysis of the brain activations in response to the three stimulus conditions (Fig. 3). For the contrast of Congruent vs. Incongruent conditions, there were no significant activation clusters (Fig. 3A), suggesting that the distance percepts are not encoded at the level of individual voxels, as these two conditions were identical in terms of the available cues while only differing in the cue congruency and, hence, the strength of the distance percept they evoke. For the contrast of Congruent vs. Fixed-DRR (Fig. 3B), a significant activation cluster containing 390 voxels and a peak at the {*x*,* y*,* z*}_MNI_ coordinates of {60, −36, 12} and *Z* = 3.29 was formed in the right hemisphere, contralateral to the stimulus direction (cluster-based MCMC simulation test, two-tailed p < 0.01). Similarly, for the contrast of Incongruent vs. Fixed-DRR conditions (Fig. 3C), a significant activation cluster with 544 voxels and a peak at the coordinates of {*x*,* y*,* z*}_MNI_ = {60, −22, 10} and *Z* = 3.39 was created in the right hemisphere (cluster-based MCMC simulation test, two-tailed *p* ≤ 0.01). In the brain volume representation, these differences extended from the posterior bank of Heschl’s gyrus (HG) to the posterior aspects of the superior temporal gyrus (STG) and planum temporale (PT). Since both of these contrasts differed primarily in the availability of the DRR cue, it can be concluded that, at the level of individual voxels, the activated areas encode primarily the DRR cue.

**Fig. 4:**
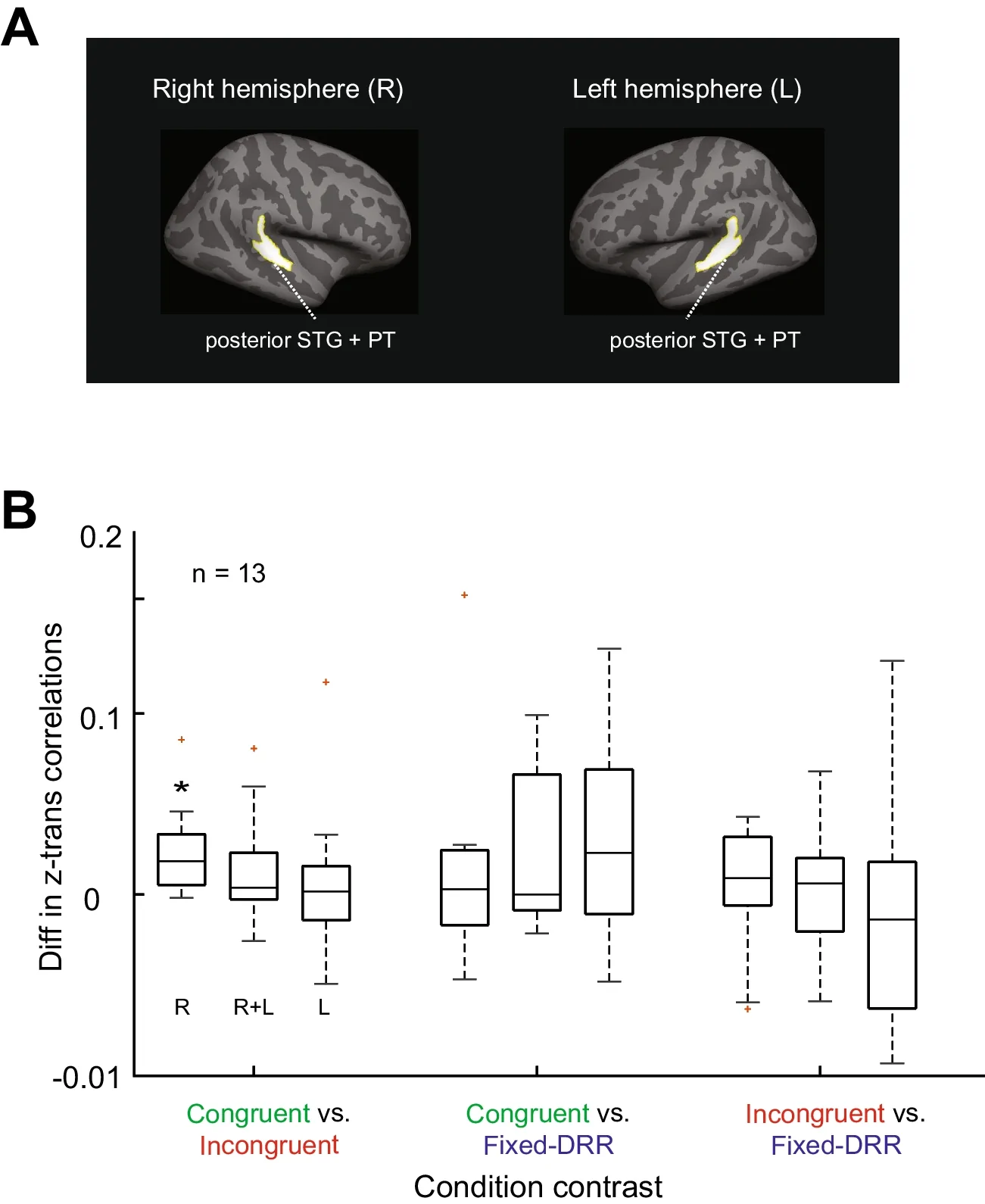
Split-half correlation multi-voxel pattern analysis (MVPA). The MVPA was performed in ROIs covering right and left PT + pSTG shown in Panel **A**. These ROIs were defined in each participant’s cortical surface reconstruction (based on the anatomical MRI) and then resampled back to their native 3D fMRI volumes. The sum of differences in *z*-transformed correlations between data from different runs for the three condition contrasts is shown in Panel **B**. (* represents *p* < 0.05 in Wilcoxon test after pos hoc Bonferroni correction for multiple comparisons)

To identify distributed patterns of activation, we performed an MVPA analysis of the fMRI data on ROIs encompassing the posterior superior temporal gyrus (pSTG) and planum temporale (PT) either in a single hemisphere (left or right) or across both hemispheres (Fig. 4A). Figure 4B shows the differences between *z*-transformed correlations for same-condition run pairs vs. different-condition run pairs for each condition contrast (triplets of bars) and different hemispheres (the bars within a triplet correspond to the right, right-and-left combined, and left hemispheres). The left-most bar in Fig. 4B shows a significant difference between Congruent and Incongruent conditions in the right hemisphere (Wilcoxon test, *w* = 88, *p* = 0.0006, effect size = 0.93), the only contrast that was identified as significant. This result suggests that the distance percepts are encoded in a distributed pattern of activation either across the pSTG + PT contralateral to the stimulus direction or across the right-hemisphere pSTG + PT. Given that a previous similar study using stimuli on the right-hand side observed activation in the left-hemisphere pSTG + PT [14], the contralateral activation alternative appears more parsimonious. Notably for the current study, the right hemisphere is the same hemisphere in which the univariate analysis identified a cluster of voxels encoding the DRR cue (Fig. 3). On the other hand, no significant effect was observed when the Incongruent vs. Fixed-DRR or the Congruent vs. Fixed-DRR conditions were compared (right-hand and middle triplet of bars, respectively). This result highlights the complementarity of the MVPA and univariate analysis approaches, as these are the comparisons for which the univariate analysis found significant clusters (Fig. 3).

Finally, a direct comparison of the MVPA and behavioral results (Fig. 2D) shows that the neural and behavioral results are mostly consistent, as both analyses find the Congruent vs. Incongruent contrast to be the strongest one, while the remaining two contrasts are weaker. However, while behaviorally even the remaining two contrasts indicate a significant difference in distance sensitivity, in MVPA they do not, possibly due to more noise in the imaging data. Thus, these results partially support the assumption that the MVPA reflects the behaviorally measured strength of the distance percept.

## Discussion

We performed behavioral and neuroimaging experiments to identify the cortical areas encoding intensity-independent distance cues vs. distance percepts. In a virtual reverberant environment, we manipulated the availability and congruency of the DRR and ILD cues for nearby stimuli simulated along the left-hand interaural axis. Behavioral results showed that distance percepts were stronger when both cues were available and congruent, supporting the hypothesis that both cues are used when listeners judge the distance of these stimuli. A univariate fMRI analysis identified areas in PT + pSTG likely encoding the DRR cue or one of its reverberation-dependent correlates, while a ROI-based MVPA over the whole PT + pSTG region found a distributed activation likely representing the integrated percept of distance. The observed activations corresponding to the cue and the percept were largely overlapping, concentrated in the hemisphere contralateral to the stimulus direction. This result suggests that hierarchical processing of auditory spatial information is occurring in this computational hub.

The current behavioral results provide direct evidence that, for lateral nearby sources in reverberation, both DRR (or its correlates) and ILD are used for intensity-independent relative distance discrimination. Previous studies suggested that ILD is the only intensity-independent distance cue for such sources in anechoic space [56, 57], while DRR might be the only cue used in reverberation [30]. Later studies suggested that, while DRR is the main intensity-independent cue for frontal sources in reverberation [31], both DRR and ILD might be used for lateral sources [14]. The current behavioral results are the first ones to provide direct evidence that both DRR and ILD are used by the listeners when the overall level is roved. However, it is still not clear which of the cues is weighted more or whether the relative contribution depends on factors like the reverberation level of the environment [58]. Moreover, the current study did not measure sensitivity in the DRR-only condition, which might be necessary to determine whether the relative cue weighting is optimal when both cues are available [59]. Similarly, the current study only focused on near distances where ILD is available. It can be expected that, at larger distances, performance would be dominated by the reverberation-related cues like DRR and the current DRR vs. ILD manipulation would not be possible. Also, the results might be different if the task was absolute, not relative, localization as the cue weighting might be different in that context. Finally, since feedback was provided in some conditions in the current study, it is theoretically possible that the participants’ responses were based on learning the cue-to-response mapping without actually eliciting the distance percept, or that the missing feedback decreased sensitivity in the Incongruent condition [60]. Overall, as these factors are unlikely to drive any of the main effects described here, the results support the conclusion that intensity-independent distance perception is robust for nearby lateral reverberant stimuli, in particular when relative distance discrimination, as opposed to absolute localization [30, 56, 57], is considered. This is because, while an absolute mapping from DRR or ILD to distance is room-dependent, the relative difference evaluation (larger DRR or ILD corresponding to a smaller distance) performed in a discrimination task is room-independent. Important for the current fMRI experiments, they also provide evidence that the auditory intensity-independent distance percepts are weaker when the cues are incongruent, corresponding to more spatially diffuse auditory objects in this condition.

The unimodal volumetric fMRI analysis identified an area in the right hemisphere extending from PT to pSTG sensitive to the DRR distance cue, while no voxels sensitive to the distance percepts were identified. This result suggests that the previous studies reporting cortical areas sensitive to intensity-independent distance variations [14, 31] most likely identified areas sensitive to the reverberation-related cue(s) like DRR, rather than the distance percept, as a unimodal fMRI analysis with similar stimuli to those used here were employed in those previous studies. Taking the current and previous studies together, it can be concluded that reverberation-related cues like DRR are encoded in the PT + pSTG region contralateral to the stimulus direction for the lateral sources, while the activation is bilateral for frontal sources. In addition, this area is also likely to encode the binaural ILD and ITD cues important for horizontal localization or motion processing [32, 46, 49, 61]. However, it is important to note that while DRR was the acoustic cue manipulated in this study, it is not likely that this cue per se is the one extracted by the cortical processing, as deconvolving the BRIR from the received stimuli would be necessary to compute DRR directly [62]. Instead, it is more likely that one of the correlate acoustic cues that vary with DRR is actually extracted in the brain, including early to late energy in the stimulus [63], interaural cross-correlation [58], amplitude modulation [22], spectral modulation [29], or temporal fluctuations in the binaural cues [64] or frequency-to-frequency fluctuations in the stimulus [8, 30].

In contrast to the unimodal fMRI results, ROI-based MVPA over the whole PT + pSTG region showed differential activation patterns in the Congruent vs. Incongruent conditions, supporting the hypothesis that the distance percepts are encoded in a distributed network that is only detectable when multiple voxels in the ROI are analyzed simultaneously [48, 49, 51]. Since the significant activation patterns were primarily observed in the right hemisphere, contralateral to the stimulus direction and overlapping with the unimodally identified DRR-sensitive regions, these results show that the PT + pSTG region encodes both the distance cues and percepts. This result is consistent with analogous evidence for sensitivity to integrated horizontal spatial percepts based on ILD and ITD cues [49], and suggests that both cue extraction and cue-independent spatial processing (i.e., encoding of the percept) occur hierarchically in this region. However, while the cue encoding is non-distributed, the percepts are encoded in a distributed network.

In general, the current findings support the idea that the PT and pSTG function as spatial computational hubs [46] that encode both spatial percepts and auditory cues. However, the details of how the different spatial cues or different integrated spatial representations of the egocentric dimensions are encoded are still largely unknown [65–67]. Future studies, ideally with more participants, need to examine both the distance representation for different environmental conditions (anechoic vs. reverberant [68–71], nearby vs. far sources [72–75], frontal vs. lateral sources [76], as well as for different types of stimuli that might use different cues [29, 77]) and the integrated representation of auditory space in all 3 dimensions. Additionally, the auditory system is highly adaptive, and different cues may govern distance perception in different acoustic environments [78]. Moreover, there is evidence that different auditory objects might be encoded already at the level of primary auditory cortex [21, 79], which might imply that the same cortical regions can subserve different functions for different objects depending on the context of the current task.

A limitation of the current study is that the intensity and ILD were always congruent in the current fMRI experiment. While it is assumed based on our previous result [14] that the intensity effect is subtracted out in the contrasts, the resulting congruency of ILD and intensity might have affected some of the current results. Similarly, the behavioral study only measured distance discrimination that could, in principle, have been accomplished by detecting changes in the cues without having a subjective externalized distance percept [80]. However, the stimuli contained natural distance cues and hence it is unlikely that the percept would not be evoked. Finally, previous evidence suggests that different activation patterns are observed in active vs passive listening [49, 81]. In the current fMRI experiments, the listeners were not involved in an active localization task, which might have resulted in weaker distance percepts or reduced responses to the stimuli. More studies are therefore needed to investigate how individual spatial features are processed across the hierarchy of auditory pathways in humans.

This study aimed to adhere to the Sex and Gender Equity in Research (SAGER) guidelines [82, 83]. However, due to inherent challenges and small sample sizes, it was not possible to follow all the guidelines. This is a limitation as the data set might be skewed by sex or race.

## Conclusions

Our results suggest that posterior human auditory cortex areas contain neural populations that are sensitive both to distance cues like DRR and the integrated percepts of auditory distance independent of intensity. The behavioral experiment further demonstrated that intensity-independent distance cues DRR and ILD are both perceptually relevant for human listeners and nearby sources located off the medial plane.

## Data Availability

The data will be made available upon request.

## Footnotes

1 Eliminating the intensity cue is challenging. Several recent distance studies tried to achieve this by systematically manipulating the emitted level to equalize the received level (e.g. [29, 84],). However, since the rate at which intensity changes with distance varies at the two ears for sources off the midline, it is in general impossible to simultaneously equalize the received level at both ears. Moreover, the received level of the direct portion and of the reverberation vary systematically with distance after such equalization, thus providing a distance cue. Finally, loudness constancy mechanisms allow people to estimate the emitted sound level in reverberation and perceive that it is being manipulated [85], thus overcoming the effect of equalization. Hence, to eliminate any chance of providing the listeners with any intensity cues, the current behavioral experiment used roving to eliminate all level cues after approximately equalizing the level at the center of the head [56].

2 One important factor limiting the studies of the neural mechanisms of auditory distance processing is that it is challenging to distinguish between the contributions of distance and intensity processing [14]. When the stimulus intensity varies with distance, physiological detectors receptive to a range of stimulus features may be activated. Although neurons in these conditions are sensitive to a specific feature at low stimulus intensities, they frequently react in a nonspecific way to loud stimuli. These nonspecific reactions are most typically observed when the stimulus is a broadband noise [86, 87] which is frequently used in investigations of auditory spatial perception. Kopco et al. [14] solved this problem by comparing responses to stimuli varying in distance and intensity to those only varying in intensity. The same approach is used here in the imaging experiment.

3 Note that in the Incongruent condition, in which no “correct” response is defined, the responses could follow the DRR or the ILD cue and the $d'_N$ expresses the extent to which a given participant followed his/her preferred cue consistently (i.e., only absolute values were considered).

4 Note that some of that difference might have come from the fact that no feedback was provided in the Incongruent condition. However, given that there was a difference between Fixed-DRR and Congruent conditions, both of which used feedback, the most parsimonious explanation consistent across all the conditions is that both cues were always used by the participants and the Incongruent condition produced a weaker distance percept even though the individual distance cues of ILD and DRR varied over identical ranges across the two conditions.

## Code Availability

The code will be made available upon request.
